# Prognostic role of resection margin in open oncologic laryngeal surgery: survival analysis of a cohort of 139 patients affected by squamous cell carcinoma^☆^

**DOI:** 10.1016/j.bjorl.2018.04.012

**Published:** 2018-06-02

**Authors:** Carmelo Saraniti, Riccardo Speciale, Salvatore Gallina, Pietro Salvago

**Affiliations:** Università degli Studi di Palermo, Dipartimento di Biomedicina Sperimentale e Neuroscienze Cliniche (BioNeC), Sezione di Otorinolaringoiatria, Palermo, Italy

**Keywords:** Laryngeal cancer, Resection margin, Local recurrence, Câncer de laringe, Margem de ressecção, Recidiva local

## Abstract

**Introduction:**

The treatment of laryngeal squamous cell carcinoma needs accurate risk stratification, in order to choose the most suitable therapy. The prognostic significance of resection margin is still highly debated, considering the contradictory results obtained in several studies regarding the survival rate of patients with a positive resection margin.

**Objective:**

To evaluate the prognostic role of resection margin in terms of survival and risk of recurrence of primary tumour through survival analysis.

**Methods:**

Between 2007 and 2014, 139 patients affected by laryngeal squamous cell carcinoma underwent partial or total laryngectomy and were followed for mean of 59.44 ± 28.65 months. Resection margin status and other variables such as sex, age, tumour grading, pT, pN, surgical technique adopted, and post-operative radio- and/or chemotherapy were investigated as prognostic factors.

**Results:**

45.32% of patients underwent total laryngectomy, while the remaining subjects in the cohort underwent partial laryngectomy. Resection margins in 73.39% of samples were free of disease, while in 21 patients (15.1%) anatomo-pathological evaluation found one of the margins to be close; in 16 subjects (11.51%) an involved resection margin was found. Only 6 patients (4.31%) had a recurrence, which occurred in 83.33% of these patients within the first year of follow-up. Disease specific survival was 99.24% after 1 year, 92.4% after 3 years, and 85.91% at 5 years. The multivariate analysis of all covariates showed an increased mortality rate only with regard to pN (HR = 5.043; *p* = 0.015) and recurrence (HR = 11.586; *p* = 0.012). Resection margin did not result an independent predictor (HR = 0.757; *p* = 0.653).

**Conclusions:**

Our study did not recognize resection margin as an independent prognostic factor; most previously published papers lack unanimous, methodological choices, and the cohorts of patients analyzed are not easy to compare. To reach a unanimous agreement regarding the prognostic value of resection margins, it would be necessary to carry out meta-analyses on studies sharing definition of resection margin, methodology and post-operative therapeutic choices.

## Resumo

**Introdução:**

O tratamento do carcinoma de células escamosas de laringe necessita de uma estratificação precisa do risco, para a escolha da terapia mais adequada. O significado prognóstico da margem de ressecção ainda é motivo de debate, considerando-se os resultados contraditórios obtidos em vários estudos sobre a taxa de sobrevida de pacientes com margem de ressecção positiva.

**Objetivo:**

Avaliar o papel prognóstico da margem de ressecção em termos de sobrevida e risco de recorrência de tumor primário através da análise de sobrevida.

**Método:**

Entre 2007 e 2014, 139 pacientes com carcinoma de células escamosas de laringe foram submetidos à laringectomia parcial ou total e foram acompanhados por um tempo médio de 59,44 ± 28,65 meses. O status de margem de ressecção e outras variáveis, como sexo, idade, grau do tumor, pT, pN, técnica cirúrgica adotada e radio- e/ou quimioterapia pós-operatória, foram investigados como fatores prognósticos.

**Resultados:**

Dos pacientes, 45,32% foram submetidos à laringectomia total, enquanto os demais foram submetidos à laringectomia parcial. As margens de ressecção em 73,39% das amostras estavam livres, enquanto em 21 pacientes (15,1%) a avaliação anatomopatológica encontrou uma das margens próxima e 16 indivíduos (11,51%) apresentaram margem de ressecção comprometida. Apenas seis pacientes (4,31%) apresentaram recidiva, o que ocorreu em 83,33% desses pacientes no primeiro ano de seguimento. A sobrevida doença-específica foi de 99,24% em um ano, 92,4% em três anos e 85,91% em cinco anos. A análise multivariada de todas as covariáveis mostrou um aumento na taxa de mortalidade apenas em relação à pN (HR = 5,043; *p* = 0,015) e recidiva (HR = 11,586; *p* = 0,012). A margem de ressecção não demonstrou ser um preditor independente (HR = 0,757; *p* = 0,653).

**Conclusões:**

Nosso estudo não identificou a margem de ressecção como fator prognóstico independente; a maioria dos artigos publicados anteriormente não tem escolhas metodológicas unânimes e as coortes de pacientes analisados não são fáceis de comparar. Para chegar a uma concordância unânime em relação ao valor prognóstico da margem de ressecção, seria necessário fazer metanálises em estudos que compartilham a definição da margem de ressecção, metodologia e escolhas terapêuticas pós-operatórias.

## Introduction

In Italy, laryngeal squamous cell carcinoma (LSCC) has a yearly incidence of 7 cases per 100,000 inhabitants (AIOM 2016) and, according to data reported by ISTAT, the Italian Institute of Statistics, 1548 patients died of LSCC in 2013. Most of these patients were aged between 60 and 80 years, and the ratio of men/women ranged from 4:1 to 20:1, based on the case histories considered.^1^

To date, the 5 year relative survival rate in Italy is 68.9% (67.7–70.2%), higher than the European average (58.9%) which is, however, significantly affected by geographical variability, and particularly by a lower survival rate observed in Eastern European countries.

Several risk factors are involved in LSCC pathogenesis, the main two undoubtedly being cigarette smoking and alcohol consumption.^2^

The treatment of LSCC needs accurate risk stratification, in order to choose the most suitable therapy, foreseeing all possible clinical outcomes. Moreover, knowing post-surgical prognostic factors might positively affect post-operation strategies, increasing patients’ survival rate.^3^

More specifically, the prognostic significance of resection margin (RM) is still highly debated,^4^ considering the contradictory results obtained in several studies regarding the survival rate of patients with a positive RM, especially after undergoing adjuvant radiotherapy.^5^, ^6^, ^7^

The main objective of this study was to evaluate the prognostic role of RM in terms of survival and risk of recurrence of primary tumour (T) through survival analysis.

## Methods

We performed a retrospective collection of data regarding 139 patients affected by LSCC, admitted and treated in our department between January 1st 2007 and April 30th 2014. Approval for this retrospective study had been obtained from the local ethical committee (Approval No. 11/2017).

All patients underwent a complete clinical evaluation, including fibre-optic video-laryngoscopy, routine blood tests, pulmonary function testing, radiography and/or CT scan of the chest, and CT scan of the neck with and without contrast.

The diagnosis of LSCC was confirmed with a biopsy performed during suspension microlaryngoscopy. Subjects excluded from our study were those either treated with transoral robotic surgery or with known distant metastasis, or with nonsquamous cell malignant tumours, or those who could not undergo the complete surgical procedure.

The cancer staging employed was the TNM criteria approved by the American Joint Committee on Cancer (AJCC) (2010).^8^

Surgical indications for open partial horizontal laryngectomies (OPHL Type I, IIa and IIb) or total laryngectomy involved patients with LSCC cT_1–4a_N_0–2c_M_0_, who had agreed to be surgically treated; in 134 (96.4%) patients, laryngectomy involved mono- and bilateral selective neck dissection (SND) levels II, III and IV. All patients who had undergone partial surgery, and who experienced recurrence, underwent salvage total laryngectomy. Out of 71 subjects who had indications for adjuvant therapy, 8 did not receive it because of health contraindications (e.g. heart, liver, kidney disease, etc.).

Reports on histopathological findings for each sample obtained during surgery included an accurate macroscopic description, specifying the anatomical site of sampling, dimensions and features of each sample, location of the tumour with a description of anatomical structures involved, a description of the SND procedure, if carried out, specifying number and size of lymph nodes found, and the involvement, if any, of surrounding anatomical structures, such as submandibular gland, sternocleidomastoid muscle and jugular vein.

Diagnostic/microscopic features also included histological type, grading, and size of tumour; the presence or absence of vascular perineural invasion was also marked, as well as infiltration of specific anatomical structures for the various sites.

The evaluation of RMs, suitably folded and sutured, was carried out on samples obtained through exeresis. Margins were classified as “free” (no tumour at or close to the margin), “close” (tumour less than 5 mm from the cut margin), or “involved” (tumour at the cut margin).^9^ An involved or close margin was considered positive; a free margin was classified as negative.

The variables reported in the dataset for each patient were sex, age, tumour grading, pT, pN, surgical technique adopted, status of RM, post-operative radio- and/or chemotherapy; dates of surgical interventions, detection of recurrence, last check-up, and death caused by the tumour under observation were also included.

Age was used as mean and standard deviation. Other categorical variables were expressed as figures and percentages. Disease specific survival (DSS) was calculated using the Kaplan–Meier method. Cox proportional hazard ratio models were used to assess independent prognostic factors and for DSS. Significant factors obtained using univariate Cox proportional hazard ratio model were included in the multivariate Cox proportional hazard ratio model, except for T, which was excluded due to multicollinearity. A value of *p* < 0.05 was considered as statistically significant. Statistical analysis was carried out using STATA.

## Results

We carried out a retrospective analysis of data from a cohort of 139 patients (Table 1), 128 men and 11 women (sex ratio = 11.6:1); the age of subjects included ranged between 42 and 87 years (mean age = 63.49 ± 10.25), with a higher mean age (*t* = 2.28; *p* = 0.023) for men (64.07 ± 10.22 years) compared to women (56.81 ± 8.44 years).

**Table 1:** Summary of clinical characteristics of the cohort.

	No.	%		No.	%
*Gender*			*Age*		
M	128	92.08	<65	74	53.23
F	11	7.92	≥65	65	46.77

*pT*			*pN*		
T_1_	6	4.32	N_0_	91	65.46
T_2_	29	20.86	N_1_	19	13.66
T_3_	54	38.85	N_2b_	11	7.91
T_4a_	50	35.97	N_2c_	13	9.35
			N_x_	5	3.62

*Grading*			*RM*		
G1	20	14.38	Free	102	73.39
G2	71	34.53	Close	21	15.1
G3	48	51.09	Involved	16	11.51

*Laryngectomy type*			*Adjuvant therapy*		
OPHL Type I	47	33.81	No	66	47.49
OPHL Type IIa	24	17.27	RT	10	7.19
OPHL Type IIb	5	3.6	RT+CT	63	45.32
Total	63	45.32			

RT, radiotherapy; CT, chemotherapy.

45.32% (63/139) of the patients underwent total laryngectomy, while the remaining subjects in the cohort underwent partial laryngectomy; in particular, in 47 cases (33.81%) a OPHL Type I was performed, in 24 (17.27) a Type IIa, and a Type IIb in 5 (3.6%).

Anatomo-pathological staging showed a locally advanced tumour (T_3–T4a_) in 104 patients (74.82%), 6 cases (4.32%) of T_1_ carcinoma and 29 (20.86%) of T_2_ type. SND was carried out in 134 subjects; in 5 cases it was not deemed necessary, given the patients’ clinical features.

Resection margins in 73.39% of samples were free, while in 21 patients (15.1%) anatomo-pathological staging found one of the margins to be close; finally, in 16 subjects (11.51%) a microscopic presence of neoplastic cells was found on one of the margins of a sample obtained through exeresis.

Following a surgical procedure, no adjuvant therapy was given to 66 patients; 10 subjects (7.19%) underwent exclusive post-operative radiotherapy, while 63 (45.32%) followed a concomitant chemotherapy. In addition, among subjects with a close RM, 9 (42.85%) were given an adjuvant therapy; the same therapy was given to 13 (81.25%) out of 16 patients with an involved margin. All 43 N_+_ patients underwent post-operative radiotherapy, with only 4 cases (9.3%) not undergoing concomitant chemotherapy.

Except for 14 cases, where post-operative follow-up occurred after less than 12 months, all other patients were followed for an average period of 59.44 ± 28.65 months (range = 12–122).

Only 6 patients (4.31%) had a recurrence, which developed in 83.33% of these patients within the first year of follow-up. Of these, two had undergone total laryngectomy, 1 OPHL Type I, and 3 OPHL Type IIa. The RM was free in two cases, closes in 3 and involved in one case. Three of the patients with a localized recurrence died during the period of follow-up, 5, 11 and 23 months after diagnosis of recurrence.

Mortality in the cohort, assessed using DSS, showed 99.24% after a year, 92.4% after 3 years, and 85.91% at 5 years.

Table 2 shows the features of patients who died during our study. Six subjects (37.5%) had undergone OPHL Type I, 4 (25%) OPHL Type IIa, 6 (37.5%) total laryngectomy. In 56.25% of the cases, anatomo-pathological tests had shown lymph node metastases, while a type T_4a_ carcinoma was observed in 87.5% of subjects; on average, death occurred within 31.93 ± 18.89 months from surgery.

**Table 2:** Characteristics of patients dead during follow-up.

*N*	Gender	Age	Grading	pT	pN	Laryngectomy type	RM	RT/CT	Recurrence	Survival (months)	Cause of death
1	M	60	2	2	0	OPHL Type IIa	Free	No	No	28	Distant metastasis
2	M	62	3	3	2b	OPHL Type IIa	Free	Yes	No	42	Distant metastasis
3	M	60	3	4a	2b	OPHL Type IIa	Involved	Yes	No	38	Distant metastasis
4	M	69	3	4a	2b	OPHL Type IIa	Involved	Yes	No	44	Distant metastasis
5	F	55	2	2	2b	OPHL Type 1	Close	Yes	No	47	Distant metastasis
6	M	63	2	3	1	OPHL Type 1	Free	Yes	No	77	Distant metastasis
7	M	70	1	3	2c	OPHL Type 1	Free	Yes	No	13	Distant metastasis
8	M	51	3	3	0	OPHL Type 1	Free	Yes	No	32	Distant metastasis
9	M	57	2	3	2c	OPHL Type 1	Involved	Yes	No	36	Distant metastasis
10	M	69	2	4a	0	OPHL Type 1	Close	Yes	Yes	33	Distant metastasis
11	M	84	1	4a	–	Total	Free	No	Yes	13	Peristomal recurrence and distant metastasis
12	M	87	3	4a	0	Total	Free	No	Yes	32	Peristomal recurrence and distant metastasis
13	M	70	3	4a	2b	Total	Free	Yes	No	14	Distant metastasis
14	M	64	2	4a	2c	Total	Free	Yes	No	2	Distant metastasis
15	M	75	2	4a	1	Total	Close	Yes	No	36	Distant metastasis
16	M	72	3	4a	2c	Total	Free	Yes	No	12	Distant metastasis

With regard to pN, 37.5% of patients classified as N_2b–2c_, died during the follow-up period, as well as 10.52% of N_1_ cases and 4.39% of N_0_ cases. More specifically, one of the N_2b–2c_ patients died during the 1st year of follow-up, 3 between the 2nd and 3rd year, and 5 between the 4th and 5th year.

**Figure 1:**
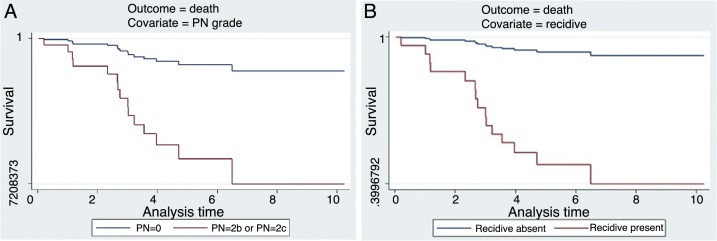
(A and B) Disease specific survival of patients with different pN (A) and with or without recurrence (B).

As shown in Table 3, a multivariate analysis of all covariates (sex, age, grading, pT, pN, type of surgery, RM status, adjuvant therapy and recurrence) showed an increased mortality rate (Fig. 1A and B) only with regard to pN (HR = 5.043; *p* = 0.015) and recurrence (HR = 11.586; *p* = 0.012). RM was not deemed as an independent predictor (HR = 0.757; *p* = 0.653); it would not, instead, appear to be linked to the risk of recurrence (*p* = 0.052).

**Table 3:** Survival analysis of the cohort.

Outcome = mortality				
Covariate (a)	Univariate analysis (HR)		Multivariate analysis (HR)	
	HR	*p*	HR	*p*
*Gender*				
M vs. F	1.563	0.666	1.211	0.863

*Age*				
1° or 2° tertile vs. 3° tertile	2.484	0.070	1.434	0.518

*Grading*				
G3 vs. G1-2	1.651	0.321	–	–

*T stage*				
T_2–3_ vs. T_1_	4.12e+08	–	–	–
T_4a_ vs. T_1_	1.09e+09	<0.0001	–	–

*N stage*				
N_2b–2c_ vs. N_0–1_	7.352	<0.0001	5.043	0.015

*Laryngectomy type*				
OPHL Type I or OPHL Type IIa or Total vs. OPHL Type IIb	1.69e+15	–	–	–

*RM*				
Positive vs. negative	1.443	0.477	0.757	0.653

*Adjuvant therapy*				
RT/CT vs. no adjuvant therapy	0.162	0.005	0.209	0.062

*Local relapse*				
Recurrence vs. no recurrence	6.591	0.004	11.586	0.012

HR, hazard ratio.

## Discussion

Deeper knowledge of the embryology, anatomy and function of the larynx has allowed the development of a wider range of surgical procedures for the treatment of LSCC, but, still today, the choices made by an ENT surgeon during an operation, or the conclusions reached by multidisciplinary teams including oncologists and radiotherapists are inevitably affected by multiple factors, some of which cannot be taken into consideration in a pre-operative phase.

Resection margins are included among these factors, and have been the focus of numerous studies, which have attempted to test their reliability from an oncologic point of view. A positive RM is now generally followed, in conjunction with a patient's clinical condition, with a widening of the exeresis and/or a post-operative radio-chemotherapy treatment, aimed at reducing the risk of localized recurrence.^10^

Notwithstanding the above, and as a demonstration of the complexity of this subject, some studies have not identified a lower risk of recurrence and/or worse survival in patients with a negative margin, compared to patients with a positive RM who had not been given an adjuvant therapy, but who had only been closely followed up.^11^, ^12^

The potential role of RM as an independent predictor of survival is still being debated, since there are no definitive data available which takes into account the heterogeneity of LSCC cases, from a staging and bio-molecular point of view.

A close observation of the main findings from analytical studies reported in Table 4 shows, above all, that the cases presented by various authors cited do not have a common denominator, in terms of type of surgical procedure and local extension of a neoplasia. Half of the quoted studies, indeed, report both partial and total (in some cases, also through endoscopy) surgical procedures on the larynx, including samples with pT_1–4_. In a few cases, RM was evaluated in patients who had undergone salvage surgery after failure of radiotherapy.^6^

**Table 4:** Literature review of the prognostic role of RM.

Authors	Year	Sample	Laryngectomy	T	R1%	R0%	Recurrence	OS	DFS	DSS	Analysis
Bradford et al.	1996	159	Partial and total	T1–T4	15.72	84.28	ns	ns	ns	–	Multi
Naudè et al.	1997	182	Partial and total	T1–T4	45.06	54.94	0.02	–	–	ns	Uni
Bron et al.	2000	69	SCPL	T1–T4	11.59	88.41	ns	–	–	<0.006	Multi
Sessions et al.	2002	200	Partial and total	T3	–	–	–	–	–	0.04	Uni
Dufour et al.	2004	118	SCPL	T3	2.54	94.92	<0.001	–	–	–	Uni
Gallo et al.	2004	253	SCPL	T1–T4	15.82	84.18	0.06	ns	–	–	Multi
Yu et al.	2006	65	Partial and total	T3	–	–	–	ns	–	–	–
Sun et al.	2009	63	SCPL e TCHEP	T1–T4	17.46	82.54	0.028	ns	–	–	Multi
Liu et al.	2009	221	Partial and total	T1–T4	17.65	82.35	–	0.015	0.001	–	Multi
Soudry et al.	2010	29	Partial and total	T1–T4	41.37	58.63	–	0.035	ns	–	Multi
Karatzanis et al.	2010	1314	Partial and total	T1–T4	9.3	90.7	–	<0.001	–	–	Multi
Liu et al.	2013	183	Partial	–	–	–	–	<0.05	–	–	Multi
Zhang et al.	2013	205	Partial and total	T1–T4	15.1	84.9	–	<0.001	–	–	Multi
Page et al.	2013	175	SCPL	T1–T3	9.14	90.86	ns	0.0001	–	–	Multi
Basheeth et al.	2014	75	Total	T1–T4	16	84	<0.001	0.03	–	0.05	Multi
De Virgilio et al.	2016	35	Total	T1, T2	0.85	99.15	–	–	–	ns	Multi
Eskiizmir et al.	2017	85	Partial and total	T3, T4	12.9	87.1	ns	ns	ns	ns	Multi

R1, positive margin; R0, negative margin; SCPL, supracricoid partial laryngectomy; TCHEP, tracheocricohyoidoepiglottopexy.

A prevalence of positive RM was seen between 1% and 45%, ranging, in most cases, between 10% and 17%, a percentage only a bit lower than the 26.6% found in our cohort.

Recurrence was not acknowledged by all authors as connected to RM, regardless of the type of surgical procedure adopted. Out of 9 studies where this connection was analyzed, five did not find any statistical significance, while in one case this was found only with regard to type T_3–4_ tumours, not in initial or intermediate stages^13^; analogously, our study could not find a statistically valid analysis either (*p* = 0.052).

It is, however, worth mentioning that, among our patients, the only two cases of a localized recurrence on a free margin occurred in patients who had undergone total laryngectomy for T_4a_ (SND was not performed in one case), not followed by adjuvant therapy, given the patient's age, >80 years. Since it was peristomal recurrence, similar to what was described by Basheeth et al., the RM may have had no relevance.^14^

With regard to the survival analysis performed by many authors, calculating curves for overall survival (OS), DSS and disease free survival (DFS), again there is no unanimous interpretation regarding the prognostic significance of RM. About half of the authors found no statistically significant difference in survival curves between subjects with positive or negative RM.^5^, ^15^, ^16^, ^17^, ^18^, ^19^, ^20^ Others, on the contrary, such as Karatzanis et al. and Page et al., as corroborated by the high statistical significance observed (*p* ≤ 0.0001), identified RM as an independent prognostic factor.^21^, ^22^, ^23^

Our study would side with the former group, even if only regarding DSS, since OS could not be assessed, as it was not possible to properly collect the information needed for this subtype of survival curve. Regarding DSS, only Bron et al. and Sessions et al. found a statistically significant difference between positive and negative RMs, even if studying two entirely different cohorts (the former consisting of patients who had undergone supracricoid partial laryngectomy (SCPL) for T_1–4_, the latter including also patients who had undergone total laryngectomy, but only for T_3_ tumours).^24^, ^25^

Some authors opted to analyze the ratio between margin and DFS, that is to say, the time elapsed between surgical procedure and neoplastic recurrence; only Liu et al. observed a significant effect (*p* < 0.05) of RM, related with the abovementioned survival rate.^26^

According to the results of our statistical analysis, coinciding with most of the studies cited, pN resulted an independent prognostic factor, with 37.5% of N_2b–2c_ patients having died during the period of follow-up (HR = 5.043; *p* = 0.015).

As well as the retrospective nature of the study, which may not include certain confounders which could influence the outcomes, our work contains other weak points. First of all, the lack of significance of certain covariates could be influenced by the cohort size and the relatively few number of events (deaths) observed; however, as showed in Table 4, it appears evident that about half of the authors cited reported smaller samples size. Secondly, even if literature is full of studies which assess the role of RM as prognostic factor including patients who underwent open but also transoral laryngeal surgery, we believe that survival analysis data about a single technique need to be provided because the choice of surgery is generally influenced by TNM and might indirectly affect the prognosis.

Discrepancies observed on comparing data available from literature with the results obtained from the analysis of our cohort of patients do not allow us to draw definitive conclusions regarding the relationship of RM with the prognosis of patients affected by LSCC.

This is largely explained by a series of critical methodological points, intrinsically connected with defining and interpreting RMs. Evaluation of RMs is the arrival point of a multi-level process involving several professionals, and including several aspects in the oncological field. These aspects, in turn, may be affected by multiple variables, which might not always be taken into account, and which are not, above all, explicitly reported in scientific studies.

## Conclusions

Our study did not recognize RM as an independent prognostic factor (HR = 0.757; *p* = 0.653); most previously published papers lack unanimous, methodological choices, and the cohorts of patients analyzed are difficult to compare, due to different staging phases, and type of laryngectomy carried out, given, unlike other authors, particularly strict selection criteria.

To reach a unanimous agreement regarding the prognostic value of RMs, therefore, it would be necessary to carry out meta-analysis on studies rigorously overlapping, with regard to definition, methodology and post-operative therapeutic choices. Another possibility which should be considered is the search for genetic markers in a margin, to help predict risk of recurrence, and/or patient survival more accurately, thus having a reliable tool, for more effective, post-operative management.^27^

## Conflicts of interest

The authors declare no conflicts of interest.
